# Evaluating cardiac function with chest computed tomography in acute ischemic stroke: feasibility and correlation with short-term outcome

**DOI:** 10.3389/fneur.2023.1173276

**Published:** 2023-07-05

**Authors:** Jie Bao, Chen Wang, Yimeng Zhang, Zhuangzhi Su, Xiangying Du, Jie Lu

**Affiliations:** ^1^Department of Radiology, Xuanwu Hospital, Capital Medical University, Beijing, China; ^2^Beijing Key Laboratory of Magnetic Resonance Imaging and Brain Informatics, Beijing, China

**Keywords:** acute ischemic stroke, hydrostatic lung, cardiac function, chest CT, computed tomography, NIHSS, outcome

## Abstract

**Background:**

The outcomes of patients with acute ischemic stroke (AIS) are related to cardiac function. Cardiac insufficiency can manifest as hydrostatic changes in the lungs. Computed tomography (CT) of the chest is commonly used for screening pulmonary abnormalities and provides an opportunity to assess cardiac function.

**Purpose:**

To evaluate the correlation between hydrostatic lung manifestations on chest CT and cardiac function with its potential to predict the short-term outcome of AIS patients.

**Methods:**

We retrospectively analyzed AIS patients who had undergone chest CT at admission and echocardiogram within 48 h. Morphological and quantitative hydrostatic changes and left ventricular dimensions were assessed using chest CT. Improvement in the National Institutes of Health Stroke Scale (NIHSS) score on the seventh day determined short-term outcomes. Multivariate analysis examined the correspondence between hydrostatic lung manifestations, left ventricular dimension, and left ventricle ejection fraction (LVEF) on echocardiography, and the correlation between hydrostatic changes and short-term outcomes.

**Results:**

We included 204 patients from January to December 2021. With the progression of hydrostatic changes on chest CT, the LVEF on echocardiography gradually decreased (*p* < 0.05). Of the 204, 53 patients (26%) with varying degrees of hypostatic lung manifestations had less improvement in the NIHSS score (*p* < 0.05). The density ratio of the anterior/posterior lung on CT showed a significant negative correlation with improvement in the NIHSS score (*r* = −5.518, *p* < 0.05). Additionally, patients with a baseline NIHSS ≥4 with left ventricular enlargement had significantly lower LVEF than that of patients with normal NIHSS scores.

**Conclusion:**

Hydrostatic lung changes on chest CT can be used as an indicator of cardiac function and as a preliminary reference for short-term outcome in AIS patients.

## 1. Introduction

Although acute ischemic stroke (AIS) mostly results from direct occlusion of the carotid-cerebral arteries, cardiac abnormalities play an important role in the pathogenesis and evolution of strokes. As the second most common risk factor for cardiogenic stroke after atrial fibrillation (AF), decreased left ventricular function is a major risk factor for functional outcomes of strokes (1). Heart failure is an important independent variable influencing stroke mortality when controlling for other factors such as AF, age, and stroke syndromes (2). The identification of impaired ejection fraction may aid in timely recognition of stroke patients who are at a higher risk of early and long-term adverse outcomes (3, 4). On the other hand, sympathetic overstimulation and the release of catecholamines following an ischemic stroke can lead to toxic injury of the myocardium and weaken the myocardial contractility. High levels of catecholamines stimulate adrenergic receptors to cause systemic vasoconstriction and increase the systemic vascular resistance, subsequently elevating the ventricular filling pressure (5, 6). Cardiac function progressively deteriorates, ultimately leading to heart failure. The interaction between AIS and impaired cardiac function may accelerate deterioration in AIS patients.

In addition to decreased left ventricular ejection fraction (LVEF) and hypertensive cardiac remodeling, decreased left ventricular function can lead to elevated pulmonary artery pressure and microvascular filtration pressure in the lungs, which cause lung injury and promote fluid formation through hydrostatic mechanisms and changes in permeability (7–9). These processes culminate in hydrostatic changes in the lungs, which may be more apparent than the cardiac changes.

Computed tomography (CT) of the chest plays a valuable role in clinical screening, especially in emergency patients with unclear clinical histories (10, 11). CT was more widely used in many institutions during the pandemic for screening of COVID-19 infection (12). Although precise assessment of cardiac function with chest CT is difficult, preliminary evaluation of cardiac function based on pulmonary involvement and gross measurements of left ventricle size can be achieved, which can potentially aid in the global evaluation of and strategic decision-making for AIS patients.

This study aimed to evaluate the correlation between hydrostatic lung manifestations and cardiac changes on chest CT and the short-term outcomes for AIS patients to assess the efficacy of chest CT as a prognostic indicator.

## 2. Materials and methods

### 2.1. Patient selection

We retrospectively analyzed the medical records of AIS patients who had been admitted to our institution between January to December 2021. All patients underwent a standard set of brain CT scans (non-contrast CT alone or multimodal CT, including perfusion and computed tomography angiography) and chest CT immediately upon admission. Inclusion criteria for this study were as follows: (1) the diagnosis of ischemic stroke met the national and international criteria for acute stroke and the time of stroke onset was less than 6 hours (from the hyperacute period), and for patients who underwent echocardiography this occurred within 48 h after admission to rule out structural abnormality; (2) patients with first-ever ischemic stroke or no legacy effects of their previous ischemic stroke; and (3) patients who had received recanalization therapy (thrombolysis or thrombectomy) and no complications or adverse effects were noted. The exclusion criteria were as follows: (1) patients with chronic obstructive pulmonary disease, bilateral diffuse emphysema, pulmonary fibrosis, severe intrapulmonary infection, or lung cancer; and (2) patients with coronary stents, prosthetic heart valves, prior coronary artery bypass graft surgery, or metal artifacts in the region of the cardiac silhouette that affected image quality.

On hospital admission, demographic data [age, sex, body mass index (BMI), and laboratory examinations] were collected, and cardiovascular risk factors (smoking, arterial hypertension, diabetes mellitus, hypercholesterolemia, atrial fibrillation, and recanalization therapy) were recorded. The National Institutes of Health Stroke Scale (NIHSS) was assessed at admission (baseline) and 7 days later. The water swallow test (WST) score was performed at admission to assess the degree of dysphagia (1) for ability to swallow the water continuously, (2) for ability to swallow the water more than twice without coughing or choking, (3) for voice quality or breathing pattern change, (4) for ability to swallow the water more than twice with coughing or choking, and (5) for inability to swallow (13, 14).

### 2.2. Measurements and outcome

All chest CT examinations were performed using a 256-detector row CT scanner (Revolution CT; GE Healthcare, Milwaukee, WI, United States). Chest CT was performed using a tube voltage of 100 kV and tube current modulation. Routine reconstruction of the chest CT images included axial images of 5 mm thickness with a high resolution and soft tissue algorithm, and axial images of 0.625 mm thickness with a high-resolution algorithm. The coronal images of the lung window were also reformatted. All the images were transferred to the picture archiving and communication system (PACS) server for image reading and further analysis.

Image evaluation was performed by two experienced radiologists (with at least 3 years of experience in chest radiology). The radiologists were blinded to the clinical information and study outcomes. Dedicated monitors and workstations for image reading with multiplanar reformation capacity were used for image analysis.

**Figure 1:**
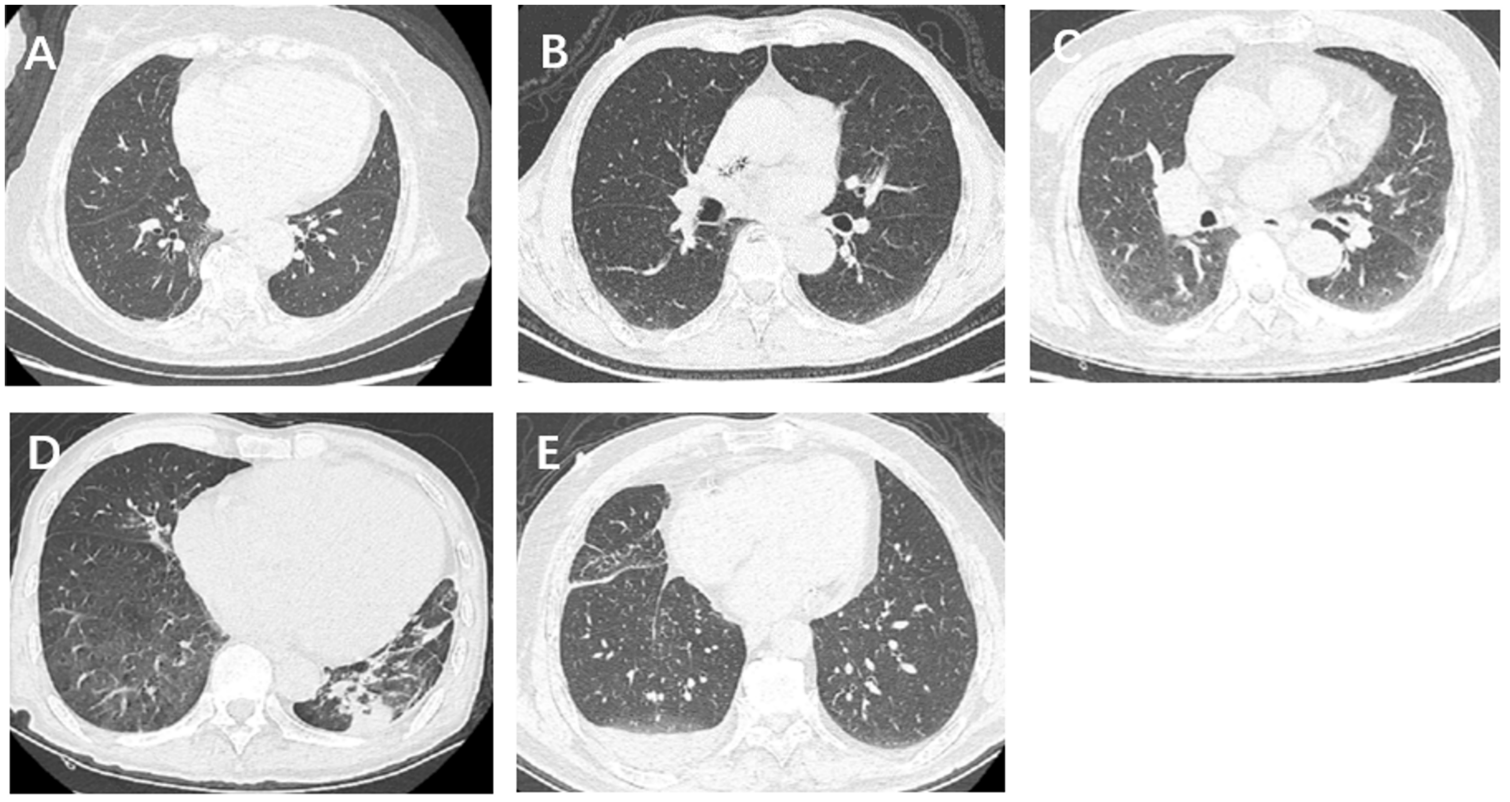
Imaging features of lungs related to hydrostatic changes, including small ill-defined opacities, interlobular septal thickening, ground-glass attenuation, airspace consolidation, and pleural effusion.

**Figure 2:**
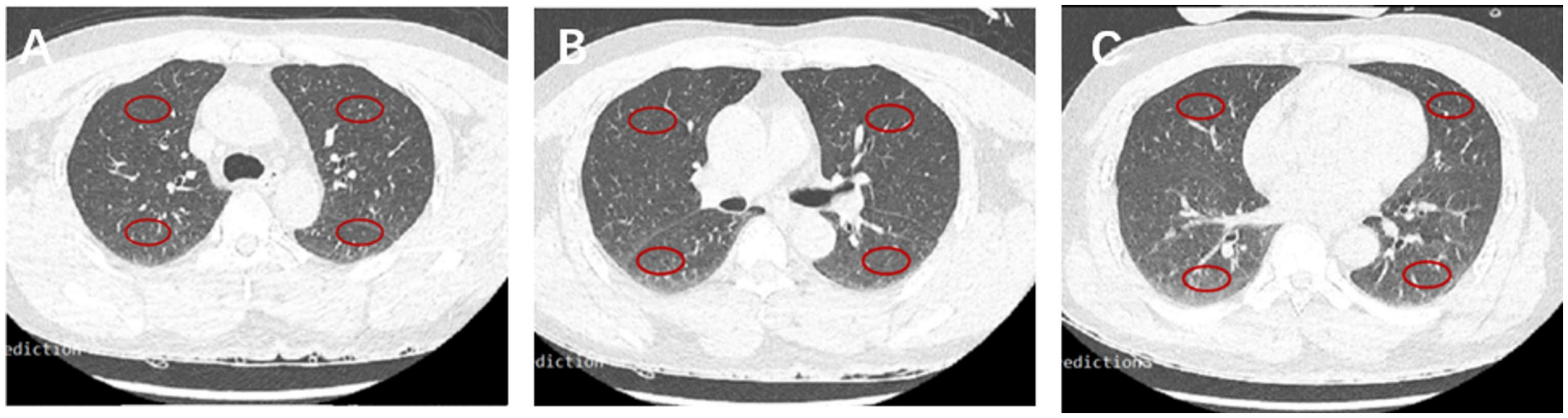
CT HU in 12 fields of the lung. Representative ROIs in different lobes in the carina **(A)**, main stem bronchi **(B)**, and bottom **(C)** levels.

The imaging features of the lungs related to hydrostatic changes and other signs were evaluated and recorded, including small ill-defined opacities, interlobular septal thickening, ground-glass attenuation, airspace consolidation, and pleural effusion (Figure 1) (15). For quantitative analysis, we used the sector method to measure the density of a peripheral area of lung parenchyma (16, 17). We manually drew regions of interest (ROIs) on the axial images in 12 regions, including the right and left upper lobes, left lingula and right middle lobe, and left and right lower lobes (size, 2 cm × 1 cm), and recorded CT Hounsfield unit (HU) of every ROI. The central vasculature, including the main and lobar pulmonary arteries, pulmonary veins, and airways, were excluded from the ROIs (Figure 2). The mean anterior/posterior lung density ratio (ΔA/P) for the patients was determined as follows: the density values in the ROIs at the peripheral one-third of the right and left anterior and posterior lung fields were arithmetically averaged.

**Figure 3:**
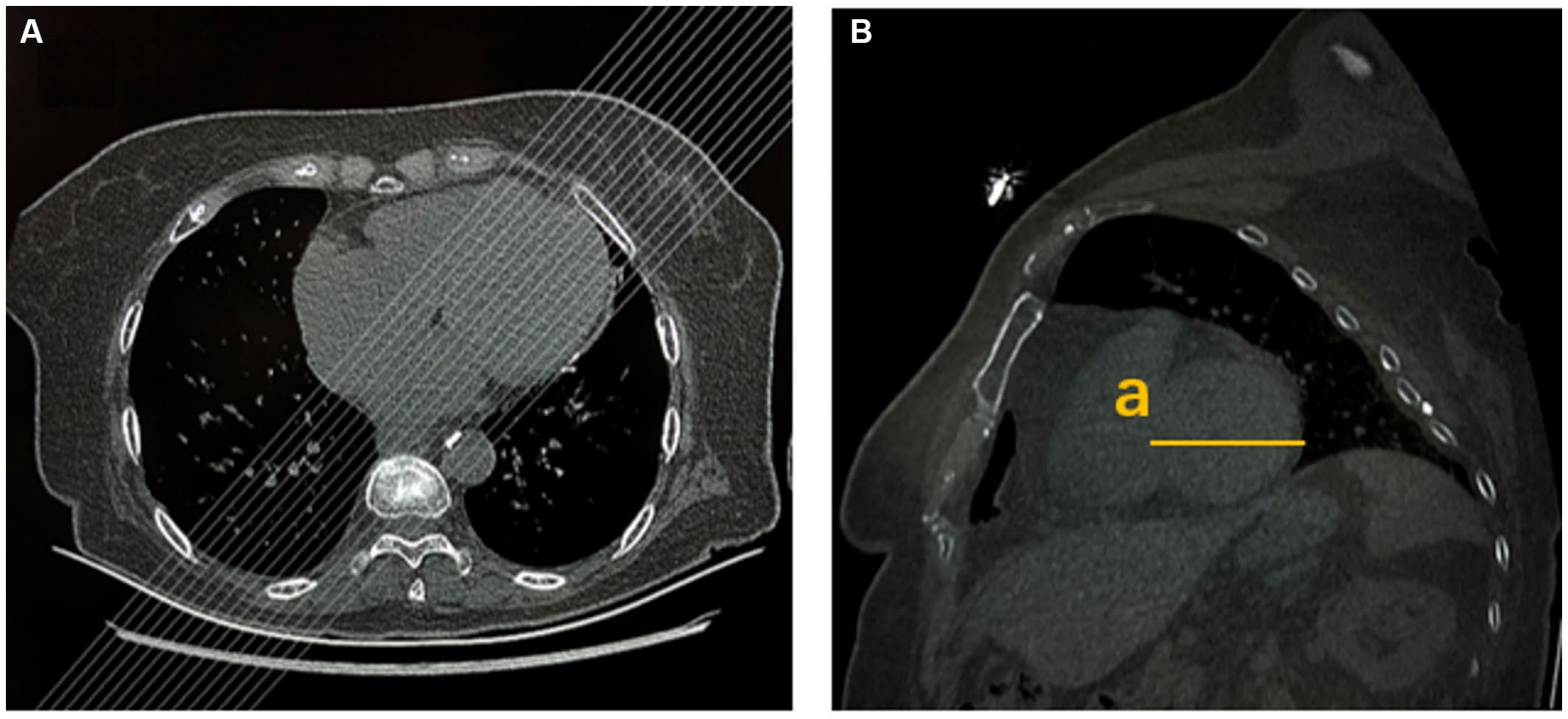
Left ventricular size was measured by chest CT. **(A)** Multiplanar reformation (MPR) after chest axial CT scans. **(B)** MPR after chest sagittal CT scans (a: maximum transverse diameter of left ventricular short axis, including interventricular septum and lateral wall).

To assess the left ventricular (LV) dimensions, short-axis images of the left ventricle were reformatted using thin-slice images on the workstation. The maximum transverse diameter of the left ventricle, including the interventricular septum and lateral wall, was measured (Figure 3). LV enlargement was defined as >66 mm deviation in men and >64 mm deviation in women from normal cardiac magnetic resonance imaging (MRI) data (18). Stroke severity was assessed using the NIHSS score at admission and 7 days later. The short-term recovery was rated as follows: not improved (NIHSS score improvement ≤30%) or improved (NIHSS score improvement >30%). Based on initial NIHSS score, stroke patients were divided into two groups, namely the mild stroke group (baseline NIHSS score ≤4) and the severe stroke group (NIHSS score >4) (19).

Measurement of cardiac dimension and function was performed with trans-thoracic echocardiography using a clinical scanner (Philips IE33 or GE Vivid E95) by certified cardiologists or sonologists. LVEF and LV dimensions were recorded for comparison with chest CT features. Left ventricular internal diameter in diastole (LVIDd) >56 mm is defined as LV enlargement in males and LVIDd >51 mm in females (20).

### 2.3. Statistical analyses

Interobserver agreement and various parameters measured using echocardiography or chest CT were assessed using the kappa coefficient statistical test. Differences in characteristics between patients with or without imaging signs were assessed by *χ*^2^ analysis or Mann–Whitney test in both mild and severe stroke groups. Frequency of hydrostatic signs in the lungs on the chest CT in AIS patients were calculated. Polyserial correlation coefficients were used to evaluate the association between continuous CT HU measurements, LV widest short-axis dimensions, and poor outcomes at 7 days. Multiple linear regression analysis was used to assess the related factors on the improvement of the NIHSS score. Receiver operating characteristic (ROC) curve analysis was performed to determine the cut-off value of CT HU measurements for differentiation between improvement of ≤30% of the NIHSS score from admission to the seventh day.

## 3. Results

### 3.1. Study population

A total of 204 patients were included in this study, with 81 and 123 in the mild and severe stroke groups, respectively. The demographic and clinical data of the two groups are presented in Table 1. Notably, the number of patients who did not show improvement was significantly lower in the mild stroke group than in the severe stroke group. Moreover, the LVEF measured by echocardiography was significantly higher in the mild stroke group. The time from stroke onset to CT, history of diabetes mellitus, acute treatment, and laboratory examinations were also different between the two groups.

**Table 1:** Demographic and clinical characteristics of the patients.

	Mild stroke (NIHSS ≤4)	Severe stroke (NIHSS >4)	*p*-value
Characteristic			
Sex (male)	60	92	0.908
Age, years	63 (56, 70)	66 (62, 71)	0.091
BMI	25 (23, 26)	25 (22, 27)	0.905
Smoking, (*n*)	48	61	0.431
Water swallow test	1 (1, 3)	1 (1, 4)	0.08
Time stroke to CT, min	198 (145, 245)	219 (166, 285)	0.035
Medical history			
Atrial fibrillation	5 (6.2%)	18 (14.6%)	0.072
Diabetes mellitus	18 (22.8%)	50 (40.7%)	0.01
Hypercholesteremia	60 (74.1%)	79 (64.2%)	0.168
Hypertension	58 (71.6%)	78 (63.4%)	0.288
Laboratory examination			
WBC	7.0 (5.7, 8.3)	8.1 (6.5, 10.1)	0.000
Neutrophils	4.3 (3.5, 5.6)	6.2 (4.6, 7.7)	0.000
NEUT%	63.8% (57.6, 72.6%)	76.6% (68.2, 83.0%)	0.000
Acute treatment			
IVT	57 (70%)	68 (55.3%)	0.04
MT	5 (6.2%)	30 (24.4%)	0.01
Location of stroke			
Left	33 (40.7%)	58 (47.2%)	0.669
Right	39 (48.1%)	53 (43.1%)	
Posterior	9 (11.1%)	12 (9.8%)	
Echocardiographic			
LVEF (%)	67 (64, 69)	63 (55, 67)	0.000
The outcome of stroke			
>30%	65 (80.2%)	82 (66.7%)	0.034
≤30%	16 (19.8%)	41 (33.3%)	

WBC, white blood cell; NEUT, neutrophil; IVT, intravenous thrombolysis; MT, mechanical thrombectomy; LVEF, left ventricular ejection fraction.

### 3.2. Morphological analysis of the lung CT

**Figure 4:**
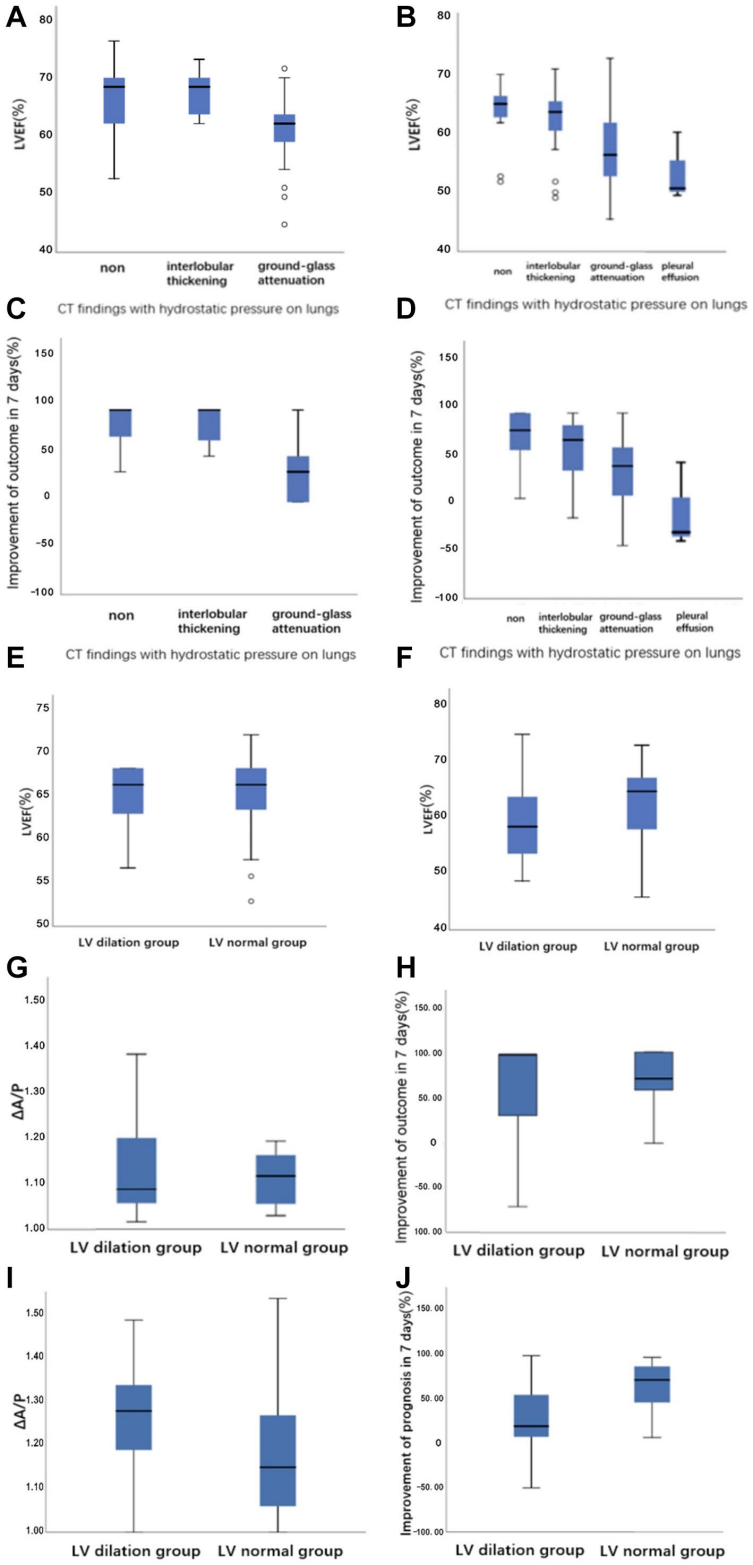
Boxplots demonstrating **(A,B)** the relationship between LVEF versus CT findings with hydrostatic pressure on lungs in the mild stroke group and the severe stroke group, respectively. **(C,D)** The relationship between the improvement of outcome in 7 days versus CT findings with hydrostatic pressure on lungs in the mild stroke group and the severe stroke group, respectively. **(E,F)** The raw data for LVEF at LV dilation and LV normal groups defined with CT image in the mild stroke group and the severe stroke group, respectively. **(G–J)** The ΔA/P and the improvement of NIHSS score in 7 days at LV dilation and LV normal groups defined with CT image in the mild stroke and the severe stroke groups, respectively.

Among the included patients, 146 (72%) had abnormal lung CT findings and tended to have a lower proportion of NIHSS improvement in both the mild and severe stroke groups, both of which were statistically significant (Table 2). The frequency of each morphological finding is presented in Table 3. The ground-glass attenuation areas in the inferior and middle lobes were the most frequent presentation of hydrostatic changes in the lungs. The number of patients with ground-glass attenuation in the lungs was significantly higher in the severe stroke group (52.8%) than in the mild stroke group (25.9%). Additionally, the patients with ground-glass attenuation areas in the lungs showed a lower LVEF measured by echocardiography (*p* < 0.001) and a lower improvement in NIHSS on the seventh day (*p* < 0.001) compared to patients without lung changes or with only interlobular septal thickening (Figures 4A–D).

**Table 2:** Baseline characteristics.

	Mild stroke (NIHSS ≤4)			Severe stroke (NIHSS >4)		
	Normal CT scan	Abnormal CT scan	*p*-value	Normal CT scan	Abnormal CT scan	*p*-value
Characteristic						
Sex (male)	26 (72.2%)	34 (75.6%)	0.734	18 (81.8%)	74 (73.3%)	0.403
Age, years	64 (55, 74)	63 (57, 68)	0.558	62 (57, 66)	66 (62, 72)	0.009
BMI	24 (23, 26)	25 (24, 27)	0.451	24 (22, 27)	25 (22, 27)	0.341
Smoking, (*n*)	21 (58.3%)	27 (60%)	0.879	11 (50%)	56 (55.4%)	0.642
Water swallow test	1 (1, 1)	1 (1, 1)	0.811	1 (1, 3)	2 (1, 4)	0.072
Atrial fibrillation	2 (5.6%)	3 (6.7%)	0.606	3 (13.6%)	15 (14.9%)	0.593
Time stroke to CT, min	170.5 (131, 214)	216 (159, 264)	0.011	188 (145, 283)	220 (174, 288)	0.275
Medical history						
Diabetes mellitus	10 (27.8%)	23 (51.1%)	0.034	5 (22.7%)	23 (22.8%)	0.996
Hypercholesteremia	26 (72.2%)	34 (75.6%)	0.734	18 (81.8%)	61 (60.4%)	0.057
Hypertension	26 (72.2%)	32 (71.1%)	0.912	16 (72.7%)	68 (67.3%)	0.622
Laboratory examination						
WBC	6.4 (5.2, 8.3)	7.1 (6.0, 8.2)	0.204	7.3 (6.1, 9.3)	8.2 (6.5, 10.3)	0.173
Neutrophils	3.9 (3.0, 5.5)	4.4 (3.8, 5.7)	0.1	4.9 (4.6, 6.9)	6.3 (4.7, 8.3)	0.123
NEUT%	61.3% (56.7, 72.1%)	64.8% (60.4, 73.6%)	0.139	69.7% (65.3, 78.9%)	76.2% (69.0, 83.3%)	0.061
The outcome of stroke						
>30%	32 (88.9%)	31 (73.3%)	0.031	20 (90.9%)	62 (61.4%)	0.008
≤30%	4 (11.1%)	14 (26.7%)		2 (9.1%)	39 (38.6%)	

**Table 3:** Frequency of each morphological finding in the mild/severe stroke group.

Morphological findings	Mild stroke (NIHSS ≤4)	Severe stroke (NIHSS >4)	*p*-value
Small ill-defined opacities	1 (1.2%)	0 (0%)	0.4
Interlobular septal thickening	23 (28.4%)	31 (25.2%)	0.613
Ground-glass attenuation	21 (25.9%)	65 (52.8%)	0.000
Airspace consolidation	0 (0%)	2 (1.6%)	0.519
Pleural effusion	0 (0%)	3 (2.4%)	0.411
None	36 (44.4%)	22 (17.9%)	0.000

### 3.3. Quantitative analysis of the lung CT

**Figure 5:**
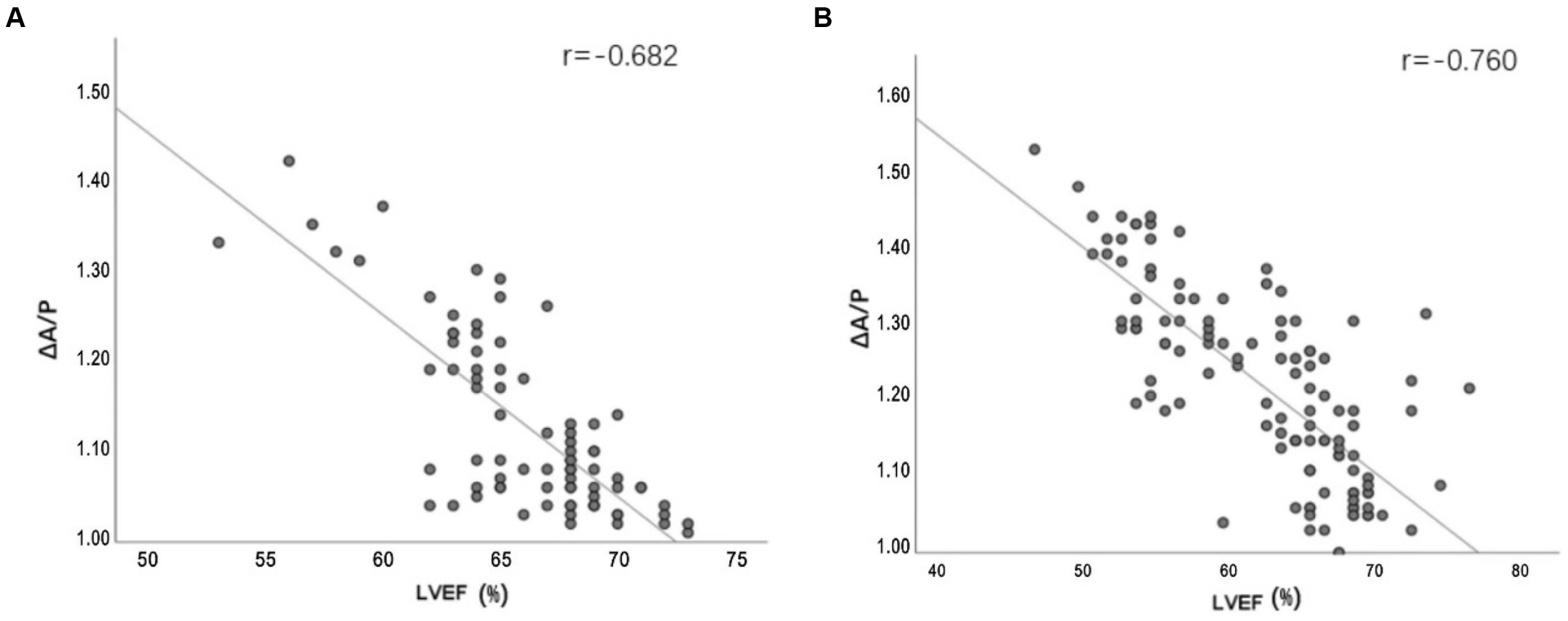
**(A,B)** Line plots demonstrating the relationship between ΔA/P measured by CT HU measurements and LVEF measured by echocardiograph in the mild stroke and the severe stroke groups, respectively.

There were strong correlations between ΔA/P and LVEF measured using echocardiography, especially in the severe stroke group (Figure 5). In the multivariate linear regression adjusted for recanalization therapy (thrombolysis or thrombectomy), NIHSS score on admission, and WST score, which had a greater impact on the improvement of outcome, ΔA/P showed a strong negative correlation with the improvement of outcome within 7 days between the two groups (Table 4).

**Table 4:** Prediction of the improvement in outcome from admission until the seventh day from quantitative analysis of lung CT and clinical factors.

Variables	β	95% CI	*p*-value
NIHSS score on admission	−0.047	(0.911, 0.995)	0.033
Water swallow test	−0.013	(0.976, 0.999)	0.030
Recanalization therapy	0.333	(0.003, 0.662)	0.048
ΔA/P	−5.518	(−6.930, −4.107)	0.000

Factors that were statistically significant (*p* < 0.05) in the multiple linear regression analysis were presented.

**Figure 6:**
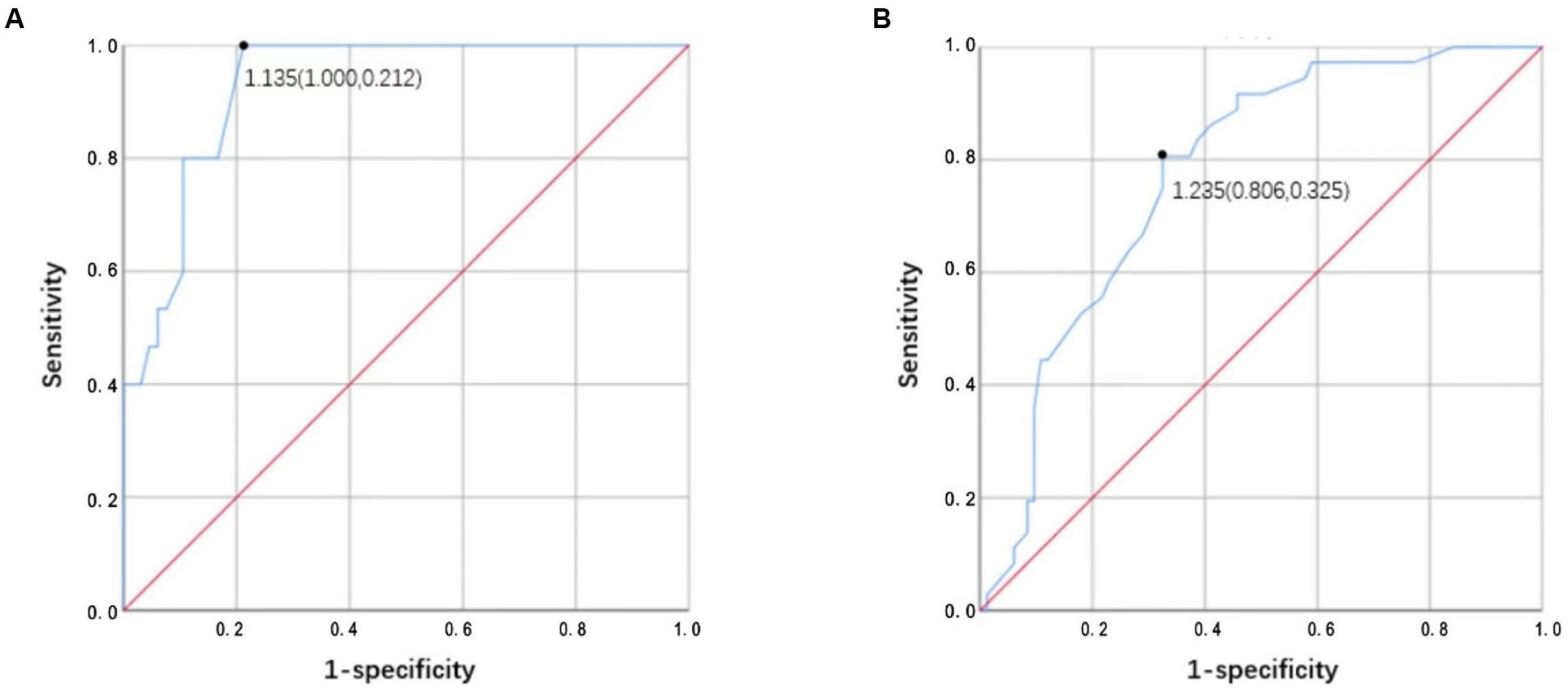
**(A,B)** Receiver operator characteristic (ROC) analysis results of ΔA/P by CT HU measurements for determining improvement <30% of NIHSS score from admission to the seventh day in the mild stroke and the severe stroke groups, respectively.

The ROC analysis results of ΔA/P for determining an improvement of ≤30% of the NIHSS score from admission to the seventh day are shown in Figure 6. The largest area under the curve in the mild stroke group was 0.928, with a cutoff value of 1.135 (sensitivity = 100% and specificity = 78.8%), and the largest area under the curve in the severe stroke group was 0.775, with a cutoff value of 1.235 (sensitivity = 80.6% and specificity = 67.5%).

### 3.4. Quantification of left ventricular size

Excellent agreement between the two radiologists was achieved for the LV measurements (kappa = 0.807). The kappa statistic showed excellent agreement between CT and echocardiography for the definition of LV enlargement in both the mild and severe stroke groups (kappa = 0.633/kappa = 0.604). As shown in Figures 4E–F, patients in the severe stroke group with LV enlargement had a significantly lower LVEF than those of patients with a normal LV (*p* < 0.001). However, this difference was not significant in the mild stroke group (*p* = 0.962). Furthermore, ΔA/P measured on CT and the improvement of NIHSS score in seven days had no correlation with LV maximum short-axis dimension in both the mild and severe stroke groups (*p* = 0.233, *p* = 0.137), but patients with LV enlargement showed a higher ΔA/P (*p* < 0.001) and a lower NIHSS improvement (*p* < 0.001) in the severe stroke group (Figures 4I,J). Similarly, a non-significant trend was observed in the mild stroke group (*p* = 0.793, *p* = 0.872), as shown in Figures 4G,H.

## 4. Discussion

Cardiac function is associated with stroke occurrence and mortality (3, 21). Preexisting cardiac failure has an adverse influence on stroke mortality independent of other known factors (2). These findings suggest the possible benefits of evaluating cardiac function in AIS patients. Echocardiography is commonly used to assess cardiac function and structure, even in emergency settings. However, it was not recommended in AIS patients according to the guidelines, unless severe heart failure presented or pre-existing severe cardiac abnormality was acknowledged (22). Other cardiac imaging modalities, including CT and MRI, are seldom performed at presentation. Chest CT has been widely used to screen hospitalized patients to prevent possible in-hospital catastrophic transmission over the last few years, although its cost-effectiveness has been debated (12, 23, 24). In addition to native pulmonary abnormalities, intrapulmonary changes related to cardiac function and dimensions can be assessed using chest CT. The development of high pulmonary capillary pressure based on decreased cardiac function results in hydrostatic gradients for fluid flux out of capillaries into the interstitial and alveolar spaces, presenting visible hydrostatic changes in the lung on CT (25). In addition, the overall LV dimension can be measured using multiplanar reformation of the heart. These findings provide an initial assessment to detect impaired cardiac function in AIS patients.

The primary findings of this study are as follows: (1) we observed a difference between improvement in early outcome among patients with and without presentation of morphological changes due to hydrostatic pressure, especially ground-glass attenuation in the lungs; (2) the ΔA/P showed a strong association with LVEF along with the improvement of outcome in 7 days. Moreover, ΔA/P had high sensitivity and specificity for differentiating patients with the potential to demonstrate >30% of baseline NIHSS score; and (3) patients from the severe stroke group demonstrating LV dilation had a higher ΔA/P and a poorer improvement in NIHSS score compared to patients without LV dilation (baseline NIHSS score >4).

Typical morphological changes due to hydrostatic pressure of the lung mainly include small ill-defined opacities, interlobular septal thickening, ground-glass attenuation, airspace consolidation, and pleural effusion. Previous studies have reported ground-glass attenuation in 25%–100% patients with hydrostatic edema. The lesions are mostly peribronchovascular owing to gravity (26, 27). In our study, patients in the severe stroke group had bilateral ground-glass attenuation areas in the inferior and middle lobes more frequently than the mild stroke group. Our study demonstrated a moderately strong correlation between hydrostatic changes in the lungs and LVEF measured by echocardiography after 48 h. Patients with ground-glass attenuation had a lower LVEF and less improvement compared to patients without morphological changes in the lung or with only interlobular septal thickening (*p* < 0.001). AIS patients showing ground-glass attenuation or more severe hydrostatic changes suggestive of decreased cardiac function at presentation showed poor short-term improvement in the NIHSS score on the seventh day.

Previous studies support our findings of quantitative analysis of CT HU measurements in AIS patients, which suggest an altered cardiac function (28–31). Rosenblum et al. (16) first used the sector method to measure the density of a peripheral area of lung parenchyma, which could detect the subtle changes in lung density including either high- or low-density lung disease and increase the likelihood that such diagnoses can be made earlier than they can from plain radiographs. Then, Slutsky et al. used the same method to relate density changes to the alteration in left ventricular filling pressure. The results showed the ratio of pulmonary density in the anterior and posterior segments (A/P) is related with heart failure (17). The dependent lung is always denser; this is attributed to the effect of gravity on the lower lobes (32). The ΔA/P may more reliably reflect changes in total lung water given the absence of additional factors that may confound density measurements. We found that ΔA/P was strongly correlated with LVEF, especially in the severe stroke group. In addition, we observed a robust association between ΔA/P and improved outcome. Evaluation of pulmonary hydrostatic changes using ΔA/P demonstrated excellent accuracy in differentiating short-term improvements of >30%, with areas under the curve as high as 0.928 in the mild stroke group and 0.775 in the severe stroke group. Moreover, ΔA/P cutoff of 1.135 in the mild stroke group and 1.235 in the severe stroke group showed high sensitivity (100% and 80.6%, respectively) and specificity (78.8% and 67.5%, respectively) for ≤30% NIHSS score improvement at 7 days. Increased ΔA/P might lead to an increased suspicion of hydrostatic pneumonia and subsequent treatments, which further affects the patients’ short-term results. Patients in the mild stroke group presented higher sensitivity and specificity, which could be attributed to the severe stroke group having a higher severity of lung disease and more influencing factors in the short term. In sum, quantitative analysis of CT HU measurements can provide first handed information of cardiac function during the acute stage of stroke. It is also useful for providing a preliminary indication of short-term outcome.

In addition to pulmonary manifestations, CT can provide information on cardiac structures that can reflect cardiac function. Previous studies have shown that LV dilation is an independent adverse predictor of cardiac function from other risk factors and is associated with an increased risk of clinical heart failure (33, 34). In our study, the results of LV evaluation using chest CT were consistent with those of echocardiography. Meanwhile, in the severe stroke group, we observed that patients with LV dilation had a higher ΔA/P and a poorer improvement in the NIHSS score than those of patients without LV dilation.

### 4.1. Advantages and limitations

The major advantage of this study was that chest CT is an easily achievable tool for excluding coexisting pulmonary conditions without a significant delay in patient management. However, due to the preliminary nature of this study, it also entailed several limitations. First, our study only included patients with hyperacute AIS; therefore, pulmonary manifestations were less affected by other factors such as subsequent infection. Evaluation of patients at other stages should be conducted in future studies. Second, chest CT can only be used for the gross evaluation of cardiac function, and the parameters are mostly indirect indicators. However, previous studies have established a linear correlation between CT HU measurements and pulmonary capillary wedge pressure measurements, as well as the New York Heart Association functional classification of heart failure (22). Third, our study was retrospective and only short-term outcomes were included in the analysis. It remains unknown whether subsequent treatment decisions based on this information can improve clinical outcomes. Therefore, further follow-up studies are required to confirm these findings.

## 5. Conclusion

A preliminary assessment of cardiac function can be achieved with qualitative and quantitative analyses of chest CT features, which can be used to predict short-term outcome in AIS patients.

## Data availability statement

The raw data supporting the conclusions of this article will be made available by the authors, without undue reservation.

## Ethics statement

Ethical review and approval was not required for the study on human participants in accordance with the local legislation and institutional requirements. Written informed consent from the patients/participants or patients/participants’ legal guardian/next of kin was not required to participate in this study in accordance with the national legislation and the institutional requirements.

## Author contributions

XD and JL conceived and designed the experiments. JB, CW, YZ, and ZS performed the experiments. JB, CW, and YZ analyzed the data. JB wrote the manuscript. All authors contributed to the article and approved the submitted version.

## Conflict of interest

The authors declare that the research was conducted in the absence of any commercial or financial relationships that could be construed as a potential conflict of interest.

## Publisher’s note

All claims expressed in this article are solely those of the authors and do not necessarily represent those of their affiliated organizations, or those of the publisher, the editors and the reviewers. Any product that may be evaluated in this article, or claim that may be made by its manufacturer, is not guaranteed or endorsed by the publisher.
